# Loss of INPP4B causes a DNA repair defect through loss of BRCA1, ATM and ATR and can be targeted with PARP inhibitor treatment

**DOI:** 10.18632/oncotarget.3307

**Published:** 2015-03-24

**Authors:** Laura R. H. Ip, George Poulogiannis, Felipe Cia Viciano, Junko Sasaki, Satoshi Kofuji, Victoria J. Spanswick, Daniel Hochhauser, John A. Hartley, Takehiko Sasaki, Christina A. Gewinner

**Affiliations:** ^1^ Department of Cancer Biology, UCL Cancer Institute, University College London, London, UK; ^2^ The Institute of Cancer Research, Signalling and Cancer Metabolism, London, UK; ^3^ Faculty of Infectious and Tropical Diseases, Immunology and Infection Department, London School of Hygiene & Tropical Diseases, London, UK; ^4^ Department of Medical Biology, Akita University School of Medicine, Akita, Japan; ^5^ Cancer Research UK Drug-DNA Interaction Research Group, UCL Cancer Institute, University College London, London, UK

**Keywords:** inositol polyphosphate 4-phosphatase type II, INPP4B, DNA damage repair, PARP inhibition

## Abstract

Treatment options for ovarian cancer patients remain limited and overall survival is less than 50% despite recent clinical advances. The lipid phosphatase inositol polyphosphate 4-phosphatase type II (INPP4B) has been described as a tumor suppressor in the PI3K/Akt pathway with loss of expression found most pronounced in breast, ovarian cancer and melanoma. Using microarray technology we identified a DNA repair defect in INPP4B-deficient cells, which we further characterized by comet assays and quantification of γH2AX, RAD51 and 53BP1 foci formation. INPP4B loss resulted in significantly increased sensitivity towards PARP inhibition, comparable to loss of BRCA1 in two- and three-dimensional *in vitro* models, as well as in *in vivo* xenograft models. Mechanistically, we discovered that INPP4B forms a protein complex with the key players of DNA repair, ATR and BRCA1, in GST pulldown and 293T overexpression assays, and INPP4B loss affects BRCA1, ATM and ATR protein stability resulting in the observed DNA repair defect. Given that INPP4B loss has been found in 40% of ovarian cancer patients, this study provides the rationale for establishing INPP4B as a biomarker of PARP inhibitor response, and consequently offers novel therapeutic options for a significant subset of patients. Loss of the tumor suppressor inositol polyphosphate 4-phosphatase type II (INPP4B) results in a DNA repair defect due to concomitant loss of BRCA1, ATR and ATM and can be therapeutically targeted with PARP inhibitors.

## INTRODUCTION

Ovarian cancer is the most common cause of gynecological cancer death and although up to 80% of epithelial ovarian cancers initially respond to chemotherapy, less than 30% of patients can be cured with currently available treatment based on 5-year survival studies [1]. Generally, genome maintenance systems detect and resolve defects in DNA ensuring that rates of spontaneous mutation are very low. In cancer, genomic instability can arise through a diverse array of defects affecting various components of the DNA-maintenance machinery, which are selectively advantageous and therefore instrumental for tumor formation and progression [2, 3]. These defects can sensitize cells towards the deleterious effects of DNA-damaging agents such as cisplatin or inhibitors of poly-ADP-ribosylation. Poly-ADP-ribose-polymerase (PARP) is a nuclear enzyme that senses DNA single strand breaks (SSBs) and is essential for base excision repair (BER). Once BER is disabled, cells rely on alternative DNA damage response (DDR) pathways, such as homologous recombination (HR), for DNA repair. Dysfunction of HR, for instance in *BRCA1*-deficient cells, presents a context in which inhibition of BER (e.g. by treating with PARP-inhibitors) is synthetically lethal [4], thus offering a therapeutic strategy for tumors with defective BRCA1. However, it is less clear how other signaling pathways such as the phosphoinositide-3 kinase (PI3K)/Akt pathway interplay with DDR and whether this can be therapeutically exploited.

Extensive studies have revealed the impact of the PI3K/Akt pathway activation on three cellular processes crucial for tumor progression: cell proliferation, cell survival and cell growth. Class IA PI3K activation gives rise to phosphatidylinositol(3, 4, 5)trisphosphate (PIP_3_) and phosphatidylinositol(3, 4)bisphosphate (PI(3, 4)P_2_) [5]. PIP_3_ and PI(3, 4)P_2_ bind to the pleckstrin homology domain of serine/threonine kinases Akt and PDK1, which results in increased cell proliferation, survival and cell growth [6]. Mechanisms to dephosphorylate PIP_3_ include the activity of lipid phosphatases PTEN and SHIP1/2, which hydrolyze PIP_3_ to PI(4, 5)P_2_ and PI(3, 4)P_2_, respectively [7, 8]. PI(3, 4)P_2_ is the main phospholipid substrate of the tumor suppressor inositol polyphosphate 4-phosphatase type II (INPP4B) which acts downstream of SHIP1/2 and produces PI(3)P, thereby terminating signaling through the PI3K/Akt pathway. Loss of INPP4B expression has been found in various human cancers with major loss in breast, ovarian and prostate cancers, as well as in melanomas [9, 10] and results in increased cell proliferation, migration and invasion. Furthermore, loss of INPP4B was shown to lead to increased signaling through the PI3K/Akt pathway [9, 11].

Recent literature suggests that Akt plays a role in modulating checkpoint responses and DNA repair. Overexpression of constitutively active Akt1, as well as PTEN loss, was found to abrogate G2 cell cycle checkpoint and Chk1 activation upon genotoxic stress [12, 13]. Studies in breast cancer cell lines and patients revealed that hyperactivated Akt promotes genome instability by repressing HR [14]. Recently, INPP4B was suggested to be a novel resistance biomarker in human laryngeal cancer, through association of high INPP4B expression levels with resistance to radiotherapy [15]. In basal-like breast cancer, DNA copy number loss of *INPP4B* was found to be associated with genomic instability and poor patient outcome [16]. However, a link between *INPP4B* status and HR function in ovarian and other human epithelial tumors has not been made but may be therapeutically beneficial. Clinical examples that take advantage of defective DNA repair in cancer therapy include single and combination treatment of germline *BRCA1/2* ovarian, breast and prostate cancers with PARP inhibitors [17]. Adopting a similar approach for INPP4B-deficient tumors may broaden the window of therapeutic applications for PARP inhibitors in a greater selection of tumors and establish INPP4B as a tumor biomarker.

## RESULTS

### *INPP4B* is frequently lost in human tumors and metastases

**Figure 1:**
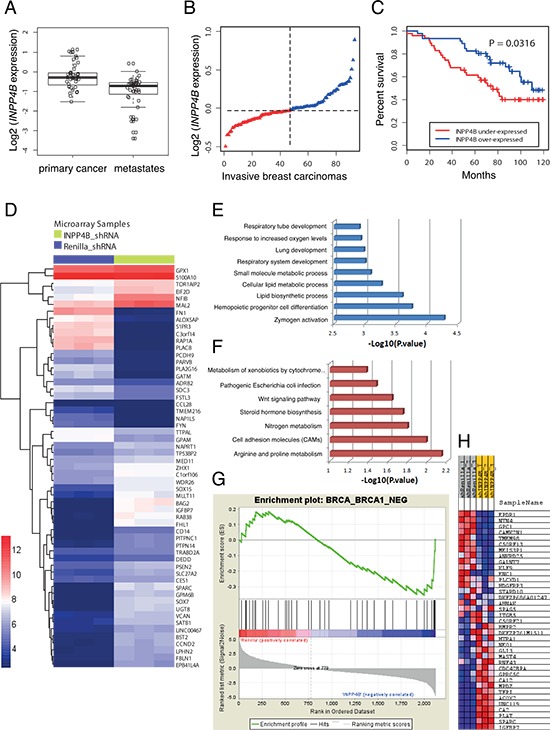
Gene expression and enrichment analysis influenced by INPP4B knockdown in MCF-10A cells **A.** Box-plots indicating significantly overall lower *INPP4B* expression in metastatic compared to primary melanomas (overall *INPP4B* loss: 48.6%). **B.** Ranked *INPP4B* expression in ductal invasive breast carcinomas (*n* = 93) and **C.** Kaplan-Meier survival curves comparing disease-free survival between cases with the lowest (< 50th percentile) vs. highest (> 50th percentile) *INPP4B* expression (*P* = 0.0316, log-rank test). **D.** Heatmap of the differentially expressed genes of MCF-10A shRNA-Renilla luciferase and shRNA-INPP4B cell pools (adjusted *p* value < 0.05). High and low levels of gene expression relative to median expression are represented by red and blue boxes, respectively. **E.** Biological processes and **F.** Kyoto Encyclopedia of Genes and Genomes (KEGG) pathways enriched in genes associated with *INPP4B* knockdown. **G.** Gene set enrichment analysis (GSEA) plot. **H.** Heatmap highlighting coordinated differential expression of the ‘BRCA_BRCA1_NEG gene signature’ in *INPP4B* knockdown cells (*P* < *p* = 0.0001). Gene expressions negatively correlated with *BRCA1* germline status in breast cancer are listed.

*INPP4B* is the target of frequent copy number loss in a variety of solid tumors including the majority of basal-like breast cancers, ovarian cancers and melanomas [9]. We performed a comprehensive analysis of *INPP4B* overall gene expression in metastatic melanomas compared to primary melanomas using the Oncomine database [18]. We found significantly lower *INPP4B* expression in metastatic melanomas compared to primary lesions (Figure 1A) corroborating earlier findings that loss of *INPP4B* expression may modulate the metastatic potential of tumors [9]. Additionally, we confirmed earlier studies demonstrating strong association of loss of *INPP4B* expression with poor overall survival in patients bearing ductal breast carcinomas (Figure 1B, INPP4B expression in invasive breast carcinoma; Figure 1C, overall patient survival) [9]. Taken together, these findings point to a critical role of INPP4B in overall patient survival and metastatic disease.

### Loss of INPP4B results in a ‘BRCA1-negative’ gene expression signature

In order to identify pathways in INPP4B-deficient tumors that may be exploited for targeted treatments, we performed a microarray gene expression analysis to compare the human mammary epithelial cell line MCF-10A stably expressing shRNA hairpins directed against INPP4B versus Renilla luciferase (control) (Supplemental Figure S1A) using the Affymetrix Human Genome U133 Plus array (~40, 000 genes, Supplemental Figure S2A to D). We identified significantly enriched gene sets in MCF-10A shRNA-INPP4B expressing cell pools compared to controls as illustrated in the heat-map (Figure 1D). Subsequent analyses of differentially expressed genes using the biological processes and Kyoto Encyclopedia of Genes and Genomes (KEGG) pathways revealed altered components in metabolic and differentiation pathways (Figure 1E and 1F). By Gene Set Enrichment Analysis (GSEA) we found that *INPP4B* knockdown cells are significantly enriched with the ‘brca_brca1_neg gene signature’ (*p* = 0.0001, Figures 1G and 1H) corresponding to differentially expressed genes in BRCA1-negative tumors. The ‘BRCA1-negative’ gene signature was validated by quantitative RT-PCR, using primers for significantly changed genes within the gene signature set (Supplemental Figure S3A). In addition, we compared our gene expression gene set with the recently established 60-gene signature for ‘BRCAness’ defined by Konstantinopoulos et al. [19], and positively correlated 71% of differentially expressed genes in MCF-10A shRNA-INPP4B with the 60-gene signature (Supplemental Figure S3B and Supplemental Table 1).

### INPP4B loss results in a DNA repair defect

**Figure 2:**
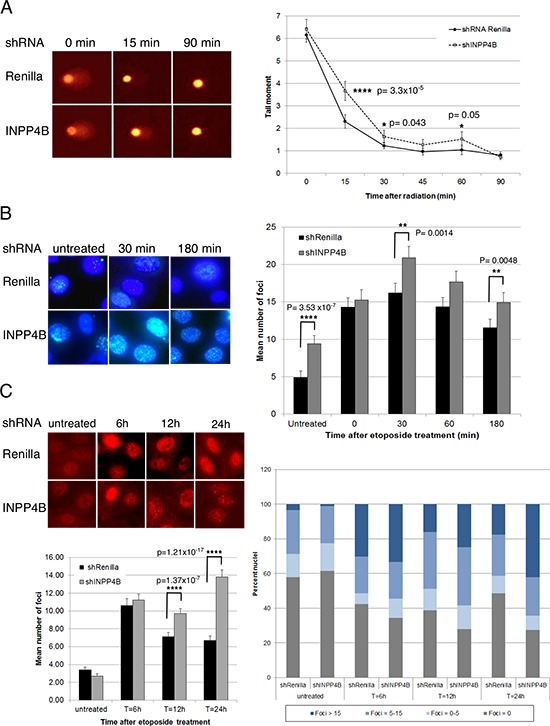
INPP4B loss in human ovarian cancer cells results in a DNA repair defect **A.** Stable Ovca429 knockdown cell pools were irradiated (30 Gy) and comet assays conducted. Tail moments were measured 0 min, 15 min, 30 min, 45 min, 60 min and 90 min post-treatment. Representative pictures are shown. **B.** Ovca429 knockdown cell pools were fixed 0min, 30 min, 60 min and 180 min after etoposide treatment and γH2AX foci quantified. Representative pictures are shown. **C.** Ovca429 knockdown cell pools were fixed 6 h, 12 h or 24 h after etoposide treatment and RAD51 foci quantified. INPP4B knockdown cell pools displayed higher percentage of nuclei containing > 15 foci per nucleus compared to the control cells. Representative pictures are shown.

Based on our findings that loss of INPP4B resembles features of BRCA1-negative tumors, we hypothesized that INPP4B deficiency may lead to defects in DNA repair. Stable knockdown cell pools of the human ovarian cancer cell lines Ovca429 and Ovca433 expressing shRNA hairpins directed against Renilla luciferase, INPP4B, PTEN and BRCA1 were generated. Cell pools over clones were chosen to best reflect heterogeneity of protein loss in the tumor environment. On average, a 50% knockdown efficiency for INPP4B was achieved (Supplemental Figure 1B). To investigate DNA repair efficiency single cell gel electrophoresis assays (Comet assays) under alkaline conditions were conducted (Figure 2A). Ovca429 knockdown cell pools were lysed 0, 15, 30, 45, 60 and 90 min after x-ray irradiation and the intensity and length of comet tails relative to head (tail moment) were analyzed after cell electrophoresis. Ovca429 shRNA-INPP4B cell pools revealed significantly increased tail moment at early time points of DNA repair compared to Renilla luciferase knockdown controls pointing to decreased DNA repair. At later time points (*t* = 45 min and *t* = 60 min) INPP4B knockdown cell pools continued to display a higher level of residual strand breaks. In contrast, we observed no abberation in olive tail moment in Ovca429 shRNA-PTEN expressing cell pools, contrary to the DNA repair deficiency associated with PTEN loss reported elsewhere (Supplemental Figure 4A) [20, 21].

Detection of Ser-139 phosphorylated histone variant H2AX (γH2AX) has emerged as a highly specific and sensitive marker for monitoring DNA damage initiation and resolution [22]. We quantified γH2AX foci formation in Ovca433 and Ovca429 cells expressing shRNA-Renilla luciferase or shRNA-INPP4B and examined *de novo* temporal and spatial distribution of DSB formation 0 min, 30 min, 60 min and 180 min after etoposide treatment (Figure 2B). INPP4B knockdown resulted in increased number and intensity of γH2AX foci compared to controls Interestingly, increased γH2AX foci were readily observed in untreated INPP4B knockdown pools pointing to an intrinsic HR defect.

H2AX plays a facilitative role in HR and has been shown to participate in RAD51-mediated suppression of DSBs generated in absence of ATR [23]. Furthermore, it was reported that retention of γH2AX foci 24 hours post-treatment with DNA damaging drugs also involved retention of RAD51 [24]. In order to assess whether RAD51 protein recruitment to sites of DNA damage is altered, Ovca429 INPP4B and Renilla luciferase knockdown cell pools were treated with etoposide and cells stained for RAD51 6 h, 12 h, and 24 h thereafter. RAD51 foci were found quantitatively and qualitatively increased in INPP4B knockdown cells (42% cells > 15 foci/nucleus) compared to controls (18% cells > 15 foci/nucleus) most prominent at 24 h (Figure 2C).

53BP1 (p53 binding protein 1) is required for p53 accumulation and G2-M checkpoint arrest in response to ionizing radiation, and 53BP1 accumulation at DSBs was reported to be dependent upon γH2AX formation [25]. Supportive of this, 53BP1 foci were found increased 6 h and 12 h after etoposide treatment in Ovca429 shRNA-INPP4B cells compared to control cells, as well as in untreated conditions (Supplemental Figure 4B).

Plo et al. demonstrated that activated Akt1 repressed HR through cytoplasmic retention of BRCA1 and RAD51 resulting in a BRCA1-deficient phenotype in breast cancer [14]. Thus, Ovca433 knockdown cell pools were fixed 0 h, 7 h and 24 h after irradiation and investigated for BRCA1 foci formation (Supplemental Figure 4C). Knocking down INPP4B in Ovca433 cells did not result in cytoplasmic retention of BRCA1 and is suggestive of a different mechanism of defective DNA repair.

### Loss of INPP4B is associated with sensitivity towards PARP inhibition

**Figure 3:**
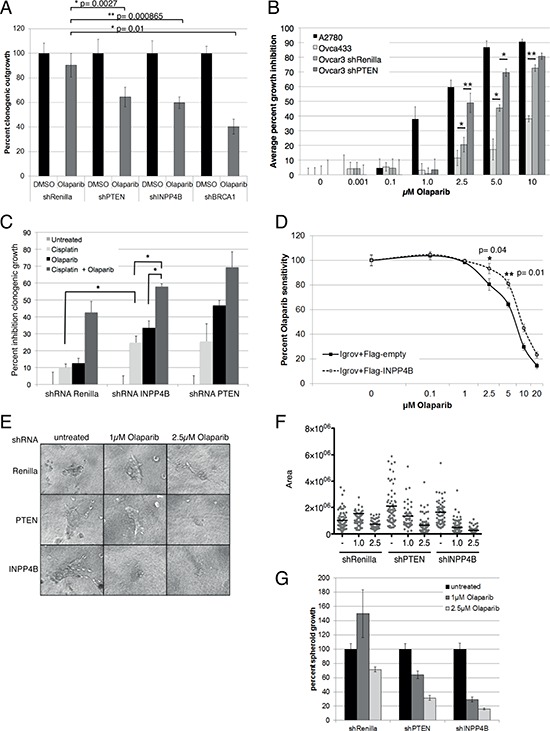
INPP4B loss sensitizes to PARP inhibitor treatment *in vitro* **A.** Ovca429 Renilla luciferase, PTEN, INPP4B and BRCA1 knockdown pools were treated continuously with 1 μM Olaparib. Percentage clonogenic growth is displayed. **B.** Olaparib dose-response growth curve of human ovarian cell lines Ovca433 (INPP4B^+/+^, PTEN^+/+^), Ovcar-3 shRNA-Renilla luciferase (INPP4B^−/−^, PTEN^+/+^), Ovcar-3 shRNA-PTEN (INPP4B^−/−^, PTEN^−/−^) and A2780 (INPP4B^−/−^, PTEN^−/−^). (*p ≤ 0.05, **p ≤ = 0.01). **C.** Clonogenic assays of Ovca429 knockdown cell pools using cisplatin alone or in combination with continuous olaparib treatment. Percent inhibition clonogenic growth displayed. **D.** Olaparib dose response curve of Igrov-1 cells (INPP4B^−/−^, PTEN^+/+^) expressing Flag-INPP4B or empty vector control. Percent growth is displayed. **E.** In three-dimensional growth assays Ovca429 Renilla luciferase, PTEN or INPP4B knockdown cells were seeded and established spheroids treated with olaparib. Representative pictures are shown. **F.** Quantification of three-dimensional assay using a linear plot, **G.** or bar graphs.

As mutations in checkpoint and DNA repair pathways are associated with cancer, tumor cells defective in HR repair with diminished or ablated BRCA1/2 gene function, show extensive DNA repair lesions and are more sensitive to PARP inhibitors [26, 27]. We next determined whether lack of competent HR due to INPP4B loss might sensitize INPP4B-deficient human ovarian cancer cells to PARP inhibition. In clonogenic assays, upon continuous treatment with PARP inhibitor olaparib, Ovca429 and Ovca433 shRNA-INPP4B cell pools displayed significantly increased sensitivity similar to BRCA1 or PTEN knockdown (Ovca429, Figure 3A; Ovca433, Supplemental Figure 5A). In proliferation assays Ovca429 shRNA-INPP4B cell pools displayed a dose-dependent and significantly increased sensitivity upon olaparib treatment compared to controls (Supplemental Figure 5B).

Next, we determined whether the observed olaparib sensitivity in Ovca429 and Ovca433 INPP4B knockdown cell pools translated into corresponding sensitivity in cell lines with varying endogenous expression levels of INPP4B or PTEN. Therefore, we characterized INPP4B and PTEN expression levels, as well as PI3K/Akt (phosphoS473 Akt) and MAPK pathway (phospho-p42/44) activation in ten different human ovarian cancer cell lines (Supplemental Figure 5C). In dose-response proliferation assays we found that A2780 (INPP4B^−/−^, PTEN^−/−^) cells demonstrated highest olaparib sensitivity followed by the INPP4B-negative Ovcar3 (INPP4B^−/−^, PTEN^+/+^). Ovca433 cells (INPP4B^+/+^, PTEN^+/+^) displayed greatest olaparib resistance. Additional PTEN loss in INPP4B-deficient Ovcar3 cells resulted in additive olaparib sensitivity similar to the A2780 cell line (INPP4B^−/−^, PTEN^−/−^) (Figure 3B; protein expression, Supplemental Figure 5D). PTEN knockdown in Ovcar3 cells resulted in additive olaparib sensitivity similar to the A2780 cell line (INPP4B^−/−^, PTEN^−/−^) (Figure 3B).

In clonogenic assays we examined growth inhibition of Ovca429 knockdown cell pools treated with cisplatin and olaparib. Cells were continuously treated with 1 μM olaparib for 6 days preceded with a single dose of 10 μM cisplatin on day 1 (Figure 3C). Ovca429 INPP4B knockdown cells showed significantly increased sensitivity to single agent cisplatin or olaparib compared to controls consistent with a DNA repair deficiency [28–31]. Combination treatment resulted in an additive effect, with significantly greater inhibition of clonogenic outgrowth in Ovca429 INPP4B knockdown pools compared to controls. Combination treatment of Ovca433 shRNA-PTEN cell pools gave similar results. In rescue experiments re-expression of Flag-tagged INPP4B in the INPP4B-deficient Igrov-1 cell line (Supplemental Figure 5E) resulted in a 2-fold increase in olaparib resistance in cell proliferation assays (Figure 3D).

In three-dimensional growth assays we investigated the effect of olaparib treatment on established spheroids from Ovca429 shRNA-Renilla luciferase, shRNA-PTEN and shRNA- INPP4B cell pools. Cells were grown on collagen layer and established spheroids were continuously treated with olaparib for three days (Figure 3E). As expected, spheroid size of mock treated INPP4B and PTEN knockdown cell pools were considerably larger than that of control cells. Upon olaparib treatment INPP4B knockdown cell pools showed significantly reduced spheroid size compared to knockdown controls (Box plot, Figure 3F; quantitation, Figure 3G); knocking down PTEN did not result in similar size reduction.

### INPP4B loss results in increased PARP inhibitor sensitivity *in vivo*

**Figure 4:**
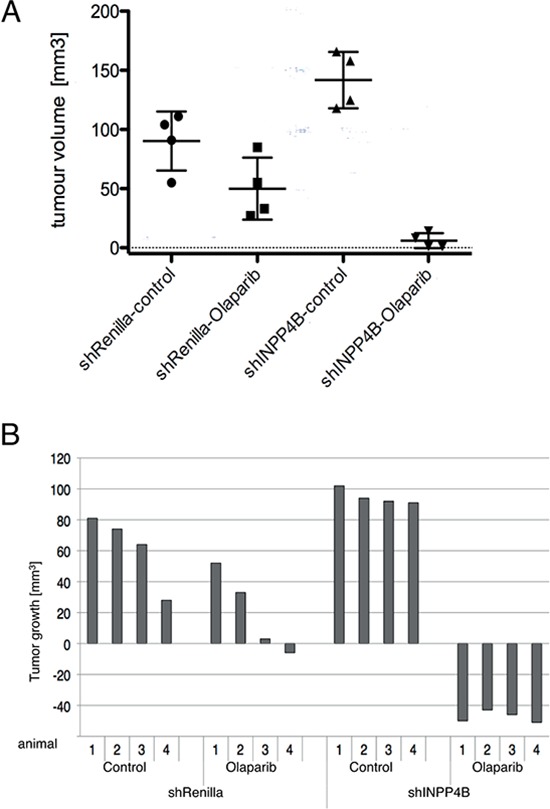
INPP4B knockdown in Ovca429 sensitizes tumor growth to PARP inhibitor treatment *in vivo* **A.** Nude mice were injected s.c. with Ovca429 INPP4B or Renilla luciferase knockdown cells. Tumor bearing animals were injected daily with olaparib or DMSO. Quantification of tumor volume is shown (error bars represent SD.). **B.** Waterfall plot of tumor volume compared to treatment start (error bars represent SD.).

Ovca429 INPP4B or control knockdown cell pools were subcutaneously injected into nude mice. Animals with established tumors were injected daily with olaparib (50 mg/kg) or DMSO. Two weeks post-treatment, mice were sacrificed and tumor volume measured and quantified. As anticipated animals injected with Ovca429 shRNA-INPP4B cells presented larger tumors compared to animals bearing Ovca429 shRNA-Renilla luciferase tumors. Upon olaparib treatment volumes of shRNA-INPP4B expressing tumors showed significant reduction compared to control treatment or shRNA-Renilla luciferase expressing tumors (Figure 4A). Waterfall plot analysis comparing tumor volumes at start and treatment end point revealed great reduction of tumor volumes in the olaparib-treated INPP4B-deficient cohort in contrast to the control cohort, which exhibited little to moderate response to treatment (Figure 4B).

### Ablation of INPP4B leads to concomitant loss of BRCA1, ATM and ATR

**Figure 5:**
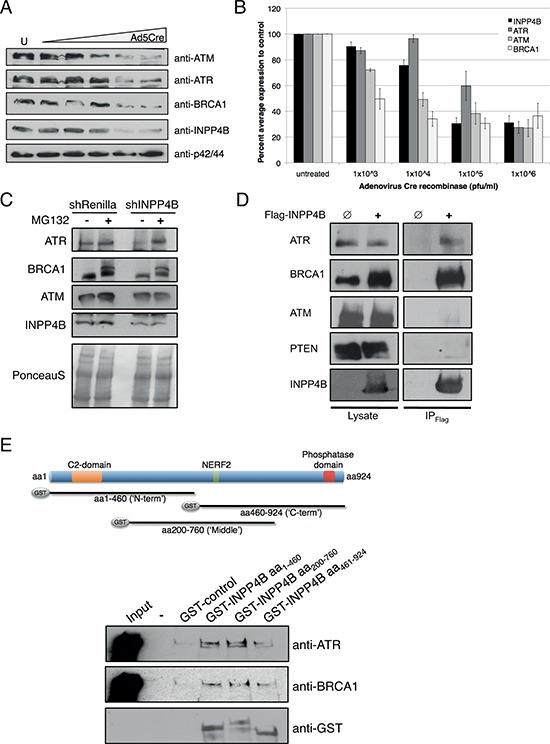
INPP4B loss results in concomitant loss of BRCA1, ATR and ATM protein levels **A.** INPP4B^fl/fl^ MEFs were infected with increasing amounts of Ad5Cre and blots probed for INPP4B, ATM, ATR, BRCA1 and p42/44 total protein levels. **B.** Quantitation of percent average protein expression to control treated INPP4B^fl/fl^ MEFs. **C.** MG132 proteasome inhibitor treatment of Ovca433 shRNA-Renilla luciferase and shRNA-INPP4B cell pools. Western blots were probed for INPP4B, ATM, ATR and BRCA1. **D.** Flag-INPP4B interacts with ATR and BRCA1 but not with ATM and PTEN in 293T immunoprecipitation experiments. **E.** GST-pull down experiments using bacterially expressed INPP4B N-terminal (aa1–460), middle (aa200-aa760) and C-terminal (aa460-aa924) fragments and ATR or BRCA1 overexpressed in unchallenged 293T cells.

We continued to examine PARP inhibitor sensitivity by ablating INPP4B expression in floxed INPP4B (INPP4B^fl/fl^) mouse embryonic fibroblasts (MEFs). INPP4B^fl/fl^ MEFs were immortalized with shRNA-p53 (INPP4B^fl/fl^ shRNA-p53), infected with Adenovirus Cre recombinase (Ad5Cre) and treated with increasing concentrations of olaparib. INPP4B loss in INPP4B^fl/fl^ MEFs upon Ad5Cre treatment resulted in significantly increased olaparib sensitivity compared to control MEFs (Supplemental Figure 5F). Western blot analysis of dose-dependent Ad5Cre infection efficiency in INPP4B^fl/fl^ shRNA-p53 MEFs demonstrated 72 h post-infection loss of INPP4B greater than 50%. Examination of BRCA1, ATR and ATM protein levels in the same lysates revealed concomitant loss of BRCA1, ATR and ATM proteins suggestive of a role for INPP4B in BRCA1, ATM and ATR stability (western blot, Figure 5A; quantitation Figure 5B).

Supporting this finding we noted a reduction of INPP4B protein in MCF-10A shRNA-BRCA1 cells (Supplemental Figure 1A and 5G). To control for a potential Ad5Cre off-target effect INPP4B, BRCA1, ATR and ATM protein levels of infected wildtype MEFs were compared to INPP4B^fl/fl^ MEFs, however no changes in protein levels were detected in wildtype MEFs (Supplemental Figure 5H).

### INPP4B affects protein stability of ATR and BRCA1

The ubiquitin-proteasome pathway mediates specific degradation of regulatory proteins and plays an important role in controlling a variety of cellular functions such as DNA repair and cell cycle control [32]. To examine the role of INPP4B loss on ATR, ATM and BRCA1 protein stability we treated Ovca433 shRNA-INPP4B and control cell pools with the proteasome inhibitor MG132, and protein expression levels were investigated (Figure 5C). Ovca433 shRNA-INPP4B cells revealed reduced ATR, ATM and BRCA1 protein levels compared to knockdown controls. While INPP4B protein expression levels remained unchanged upon treatment with MG132, expression of ATR and BRCA1 protein levels increased compared to control pools, suggestive of a role of INPP4B in ATR and BRCA1 protein stability. No change in protein expression was observed for ATM protein levels.

### INPP4B forms a protein complex with ATR and BRCA1

We further examined whether INPP4B interacts in a protein complex with the DNA repair proteins ATR, ATM and BRCA1. 293T cells expressing Flag-tagged INPP4B (Flag-INPP4B) or empty Flag vector (control) were lysed and Flag-INPP4B protein complexes immunoprecipitated. Western blot analysis demonstrated specific interaction of INPP4B with ATR and BRCA1, but not with ATM or PTEN (Figure 5D). Interestingly, we observed increased protein levels of BRCA1 in cell lysates overexpressing Flag-INPP4B compared to control lysates. GST-pull down analyses using bacterial overexpressed GST-tagged fragments of N-terminal (aa1-aa460), middle (aa200-aa760) or C-terminal (aa460-aa924) INPP4B and in 293T cells overexpressed Flag-tagged ATR or BRCA1 revealed specific interactions of ATR and BRCA1 with the N-terminal and middle fragment of INPP4B but not with the C-terminal fragment in immunoblots (overview Figure 5C, western blot Figure 5E).

## DISCUSSION

Up to 50% of epithelial ovarian cancers display defects in the DNA damage response (DDR) pathway. Recent studies have implicated Akt in modulating DDR and genomic stability [33]. Similar defects in homologous recombination (HR) have been described for loss of PTEN, although its mechanism remains controversial [12, 21]. In this study we identified a DNA repair defect using microarray analysis in INPP4B-deficient human mammary epithelial cells. Additionally, we revealed that tumors deficient in INPP4B display significant sensitization towards PARP inhibition due to concomitant loss of BRCA1, ATM and ATR. Tumors impaired in HR exhibit increased sensitivity towards platinum-based agents, the current first line treatment for ovarian cancer patients. Accordingly, INPP4B knockdown cells demonstrated increased sensitivity upon cisplatin treatment and in combination treatment with PARP inhibitor olaparib we found additive growth suppression. Olaparib has demonstrated single agent activity in breast and ovarian cancer patients with *BRCA1/2* germline mutations [34, 35], with over 40% response rate reported in patients with *BRCA1*-mutant ovarian cancer [34]. Additional fractions of patients who may benefit from PARP inhibitors have been recently identified by the Cancer Genome Atlas Research Project, which reported that 50% of the analyzed 489 serous ovarian adenocarcinomas harbor a defect in the HR pathway [24]. These results suggest that ovarian cancer patients with sporadic abnormalities in the HR pathway may benefit from treatment with PARP inhibitors. Currently, patients are being stratified for PARP inhibitor treatment by germline *BRCA1/2* mutation status, which comprise 10–15% of ovarian cancer patients. However, INPP4B loss has been found in 40% of ovarian tumors revealing the clinical significance of INPP4B and its potential use as a biomarker of PARP inhibitor response for a broadened patient subset.

At present, it is being discussed whether PARP inhibitors should be used alone or in combination with chemotherapy in clinic; however, substantial bone marrow toxicity has been reported for combination of PARP inhibitors and chemotherapy. Given different underlying defects of DNA repair may cause varying sensitivities to PARP inhibitors in sporadic cancers, in the context of tumor heterogeneity, combination treatments using PARP inhibitors together with other targeted therapies may be more effective. Juvekar et al. recently reported efficacious combination of PARP inhibitors with the dual PI3K/mTOR inhibitor NVP-BKM120 in BRCA1-related breast tumors [36]. Ibrahim et al. showed that PI3K blockade using PI3K inhibitors resulted in HR impairment and sensitization towards PARP inhibition in triple negative breast carcinomas without BRCA1/2 mutations [37]. However, we demonstrate that INPP4B loss and subsequent activation of the PI3K/Akt pathway resulted in significant sensitization towards PARP inhibition suggesting an independent role for INPP4B in HR in ovarian cancer.

**Figure 6:**
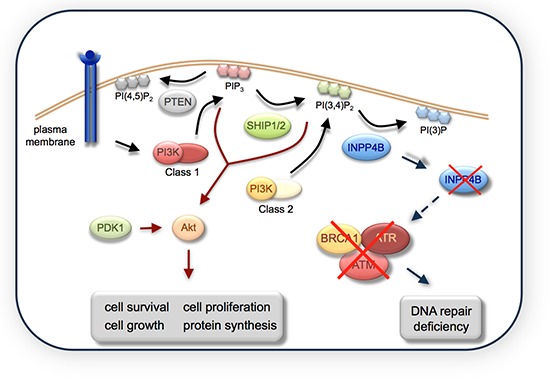
A model indicating the role of INPP4B in DNA repair INPP4B loss causes a DNA repair defect through loss of BRCA1, ATM and ATR total protein levels.

In agreement with an underlying defect in DDR we found increased accumulation of γH2AX foci in unchallenged INPP4B knockdown cell pools compared to controls. Upon genotoxic stress, induced etoposide treatment, INPP4B-deficient cells revealed an increase and retention of γH2AX, RAD51 and 53BP1 foci relative to controls indicating abnormal DNA repair and dysfunctional HR. While loss of BRCA1 function was shown to result in suppressed RAD51 foci formation, loss of ATR was linked to increased RAD51 foci formation in conjunction with increased γH2AX foci [23, 24]. Intriguingly, INPP4B loss led to concomitant loss of BRCA1, ATM and ATR total protein levels in MEFs. We also observed reduced ATR protein levels in Ovca429 INPP4B knockdown cell pools compared to controls (Supplemental Figure 5G). Additionally we noted in MCF-10A shRNA-BRCA1 cell pools decreased levels of INPP4B. Although the degree of INPP4B loss that is needed for cancer cells to down-modulate ATR and BRCA1 levels and to acquire sensitivity towards PARP inhibitors still needs to be determined, we found that INPP4B loss greater than 50% in MEFs resulted in reduced levels of BRCA1, ATM and ATR proteins. Mechanistically, INPP4B loss may potentially affect ATR and BRCA1 protein stability due to disrupted protein-protein interaction in INPP4B-deficient cells (summarized in Figure 6). In GST pull down assays we identified the N-terminal region of INPP4B (aa1–460) necessary for interaction with ATR or BRCA1. Whether this effect is a result of direct or indirect interaction of INPP4B with ATR and BRCA1, and whether catalytic activity is required for this association will need to be further elucidated. However, our studies reveal that INPP4B plays a crucial role in modulating the stability of key players of the DDR pathway and can be therapeutically exploited for cancer patients, thus providing the rationale to investigate INPP4B as a biomarker of clinical response to PARP inhibition in ovarian cancer. Thus, loss of the tumor suppressor inositol polyphosphate 4-phosphatase type II (INPP4B) results in a DNA repair defect due to concomitant loss of BRCA1, ATR and ATM and can be therapeutically targeted with PARP inhibitors.

## MATERIALS AND METHODS

### Bioinformatic analysis of *INPP4B*

Transcriptional levels of *INPP4B* were plotted in primary and metastatic tumor samples of melanoma origin using The Cancer Genome Atlas - http://tcga-data.nci.nih.gov/tcga/ [38]. Distributions of log2 median-centered signal intensities were plotted using boxplots and differential gene expression was computed using Welch two sample *t*-test. An univariate cox proportional hazard regression model [39] was applied to correlate gene expression of *INPP4B* with patient's survival in an invasive ductal breast carcinoma dataset of *n* = 93 samples [40] and the Likelihood ratio test, Wald test, and Score (log-rank) test were used to compute *P* value (*P* < 15 × 10^−4^ for all three tests). Samples obtained from survival analyses were ranked according to *INPP4B* gene expression and Kaplan-Meier survival curves were plotted for breast carcinomas with the lowest 50th versus highest 50th percentile of *INPP4B* expression giving a *P* value of 3 × 10^−2^ (log-rank test).

### Microarray

Total RNA from three independently generated RNA preparations of MCF-10A shRNA-*INPP4B* or shRNA-Renilla luciferase cells was hybridized according to standard protocol required for Affymetrix U133 Plus 2.0 arrays at the BIDMC Genomics and Proteomics Core (*n* = 3 per shRNA construct). Data pre-processing and quality control were performed in R (http://www.r-project.org/) and Bioconductor. Prior to any statistical computation, data were normalized using gc-Robust Multi-array Average (gcRMA) algorithm. Differentially expressed genes were computed by empirical Bayes (eBayes) shrinkage of standard errors toward a common value approach [41] embedded within the Limma package. *P*-values were adjusted for multiple comparisons using the false discovery rate approach [42].

Unsupervised hierarchical clustering using the Euclidean distance metric and complete linkage algorithm was used to plot the differentially expressed genes with an adjusted *P* value < 0.05. Gene ontology and pathway enrichment analyses were performed using a classical hypergeometric test and the gene ontologies or pathways with the highest negative log (10) *P* value were plotted [43]. Microarray data are available at the Gene Expression Omnibus of the NCBI.

### Cell culture

MCF-10A cells (Prof. Joan Brugge, Harvard Medical School, USA) were maintained as described elsewhere [44]. Methods to produce INPP4B^fl/fl^ mice will be presented elsewhere. Ovarian cancer cell lines were obtained from Prof. Simon Gayther (USC, USA). Stable cell lines were generated using retroviral or lentiviral hairpins and selected with 2 μg/ml puromycin. MSCV-U6miR30-PIGdeltaRI-FF2 (shRNA-Renilla luciferase), and MSCV-U6miR30-PIGdeltaRI-shRNA-PTEN were a gift from Steven Elledge (Harvard University, USA), and shRNA-BRCA1 (RHS4430–98708636) purchased from OpenBiosystems. INPP4B^fl/fl^ MEFs were infected with Adenovirus Cre recombinase (1×10^3^pfu/ml Ad5CMVCre-eGFP, Gene Transfer Vector Core, USA) overnight. For protein degradation studies, cells were treated with 1 μM MG132 for 7 h before cell lysis.

### Immunoblotting

Cells were lysed in Cell Signaling lysis buffer. Western blotting was performed with the following antibodies: Cell Signaling: INPP4B (#8450), phospho-Akt (Ser473) (#4060), Erk1/2 (#4695); Santa Cruz: PTEN (sc-6817-R), Akt 1/2 (sc-1619), BRCA1 (sc-642); Millipore: H2A.X (Ser139) (#05–636), ATM (#07–1286); Bethyl: ATR (A300–138A).

### RT-PCR

RNA was extracted using the RNeasy mini kit (Qiagen). Power SYBR Green RNA-to-C_T_^™^ 1-Step Kit (Applied Biosystem) was used with Mastercycler EP realplex real-time PCR system (Eppendorf). Primer sequences were obtained from PrimerBank, Massachusetts General Hospital, Harvard University) [45].

### Colony formation assay

Cells were seeded in 6-well plates (*n* = 3) and treated the following day. Dual drug treatment of cisplatin and olaparib (AstraZeneca, UK) was administered by adding 10 μM cisplatin on day 1 for 1 h, followed by 1 μM olaparib treatment for 6 days with media change every other day.

### Cell proliferation assay

Cells were seeded in 96 well plates (*n* = 3). Cells were subjected to olaparib treatment as indicated and media exchanged every other day. Cell growth was examined on day 3 using AlamarBlue.

### Immunoprecipitation and GST pulldown assays

293T cells were transfected with pcDNA3/FLAG empty or pEAK/FLAG-INPP4B using PEI (9002–98-6, Polyscience). 48 h post-transfection cells were lysed and lysates incubated with M2 beads. Beads were washed in TBS-T and TBS. GST pull down studies were performed as published previously [46, 47]. Input lanes represent 1% of protein lysates in pulldown assays.

### Comet assay

Comet assays were performed as described elsewhere [48]. Image analysis was performed using an inverted Nikon microscope, Luca S digital camera and KOMET software version 6.0 (Andor Technology Limited). Ovca429 and Ovca433 cells were irradiated with 30 Gy and 35 Gy, respectively. In total, 50 cells/sample, 25 cells/slide were analyzed.

### Immunofluorescence

Cells were seeded into 8-chamber well slides and irradiated (2 Gy; BRCA1) or treated with 1 μM (γH2AX) 10 μM (RAD51, 53BP1) etoposide for 1 h and fixed at indicated time points. Cells were blocked, incubated with primary antibodies (H2A.X (Millipore, #05–636), 53BP1 (Cell Signaling, #4937), RAD51 (Santa Cruz, sc-8349), in blocking buffer overnight at 4°C, incubated with secondary antibody (AlexaFluor, Invitrogen) and mounted with Fluoroshield containing DAPI. Images were captured with inverted microscope Zeiss Axio Observer Z1 and visualized using AxioVision 4.8.2.0 software.

### Three-dimensional growth culture

96-well plates were coated with 5 mg/ml rat collagen type 1 and allowed to gel at 37°C. Cells were plated on top in growth media containing 0.5 mg/ml collagen. After spheroids were formed cells were treated as indicated for 3 days. Phase-contrast microscopy was used to image 3D structures. Areas of more than 20 spheroids were measured using ImageJ (NIH).

### Xenograft experiments

Ovca429 shRNA-Renilla luciferase and Ovca429 shRNA-INPP4B expressing cells were injected subcutaneous in female NOD/SCID mice (1 × 10^7^ cells/animal, *n* = 8 each cell pool). Tumors were grown until 50 to 60 mm^3^ tumor volume then treated with 50 mg/kg olaparib daily except weekends for two weeks. Tumor occurrence and size were evaluated. All animal studies were approved by the UCL Biological Services Ethical Review Committee and licensed under UK Home Office regulations Act 1986 (Home Office, London, UK).

### Data and statistical analysis

All graphs display cumulative data representing three independent experiments and standard error was used to display data variability, unless otherwise stated. For statistical analyses we used the two-tailed, paired Student's *T*-Test.

## SUPPLEMENTAL FIGURES AND TABLE




