# Supplementary material for: Loss of INPP4B causes a DNA repair defect through loss of BRCA1, ATM and ATR and can be targeted with PARP inhibitor treatment
Source: Oncotarget. 2015 Mar 24;6(12):10548–62. doi: 10.18632/oncotarget.3307 (PMC4496374; doi:10.18632/oncotarget.3307)
Supplement: Supplementary file 1 [file oncotarget-06-10548-s001.pdf]

SUPPLEMENTAL FIGURES AND TABLE

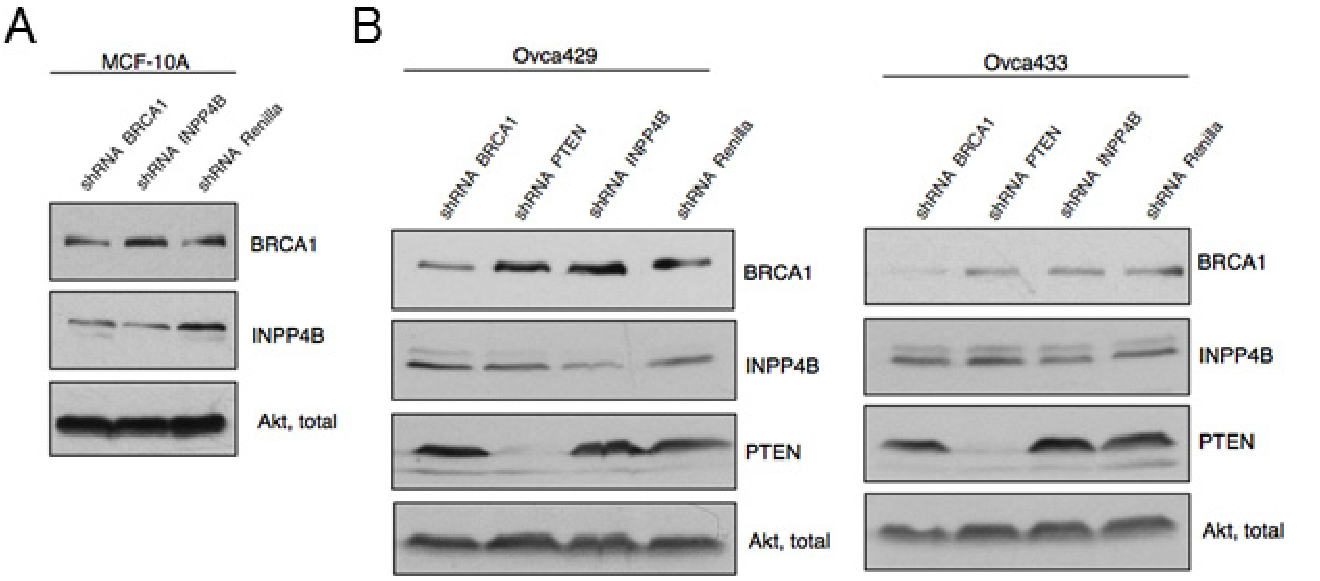

Supplemental Figure S1: Western blot analyses of ovarian cancer cell lines and stable knockdown cell pools. A. Analysis of INPP4B and BRCA1 knockdown in the human mammary epithelial cell line MCF-10A. **B.** Analysis of Ovca429 and Ovca433 knockdown cell pools. Cell lysates of stable Ovca429 and Ovca433 shRNA hairpin expressing cell pools were examined for INPP4B, PTEN and BRCA1 knockdown compared to shRNA-Renilla luciferase (control) expressing cell pools. Total Akt1/2 protein levels served as loading control.

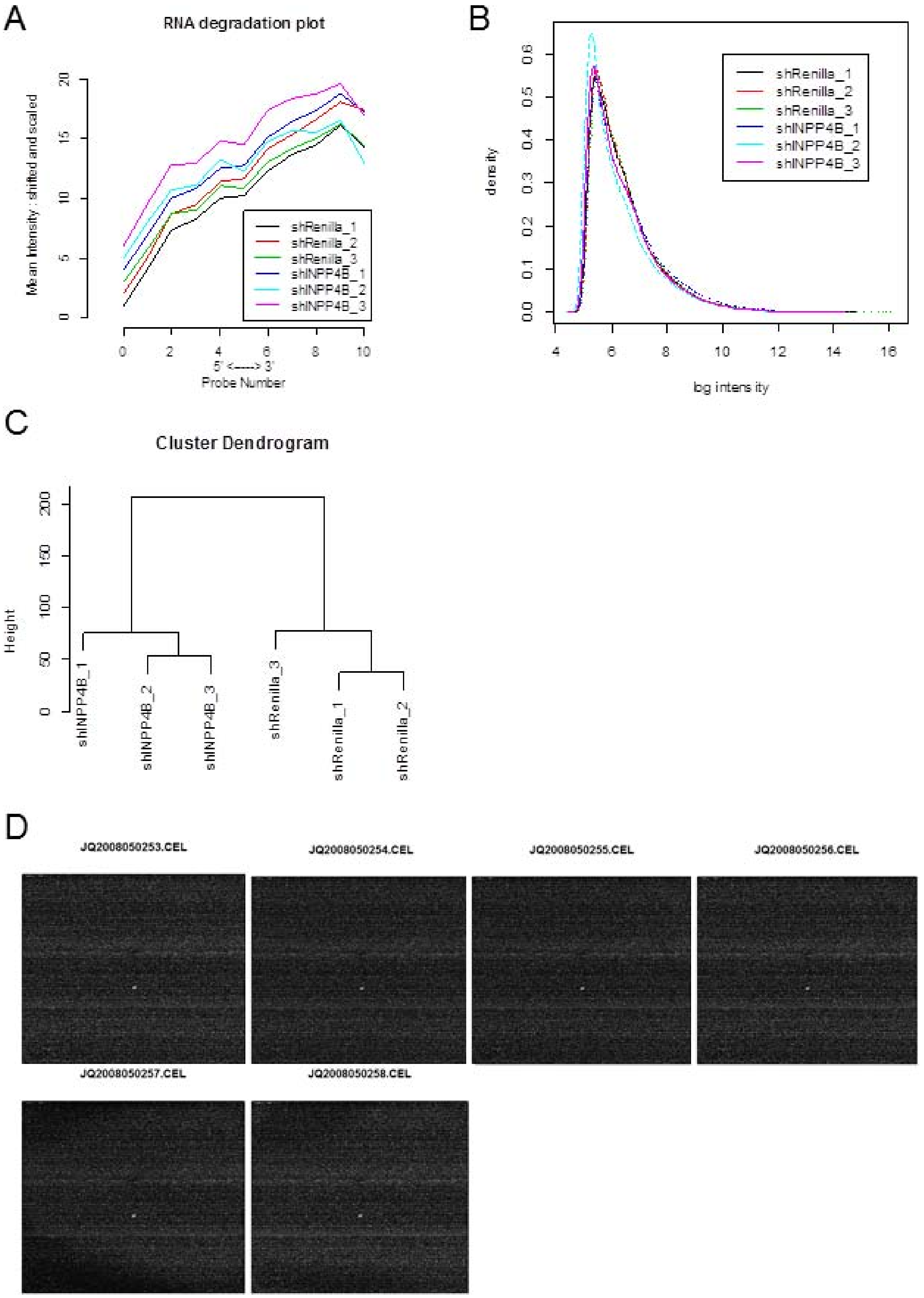

Supplemental Figure S2: Qualitative analysis of the microarray experiments (*n* = 6) that were used to assess the transcriptional changes of INPP4B or Renilla knockdown in triplicate. A. RNA degradation plots indicating the relative intensities of individual probes ordered from 5' to 3' averaged over all probe sets. **B.** Scatter plots of the log-intensity values of the genes across all microarray experiments. **C.** Unsupervised hierarchical clustering across the 54,675 gene probes that were included in the microarray platform indicating good clustering among the replicate samples. **D.** Image plots of the arrays that were used in this experiment indicating no major artifacts.

A

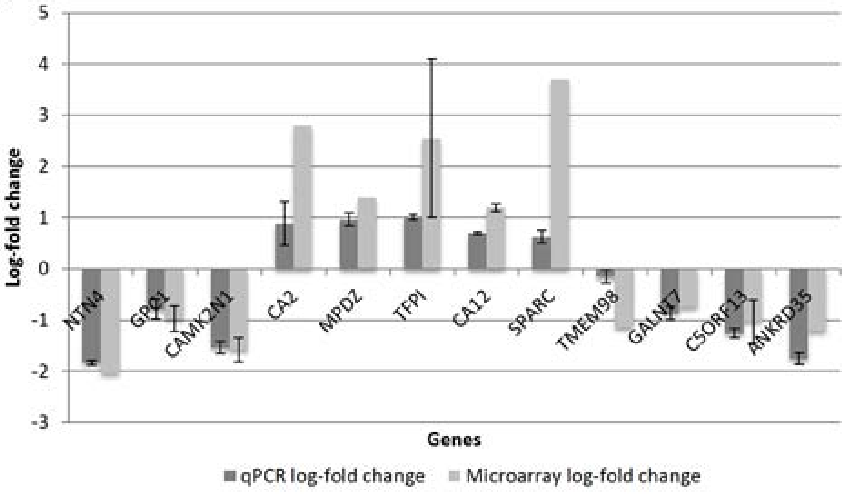

Gene name	Log (2) Fold Change	Standard Deviation	P Value	Adjusted P Value
ANKRD25	-1.21		2.02E-05	1.57E-03
C5ORF13	-1.02	0.42	5.04E-04	1.35E-02
CA12	1.20	0.08	4.20E-05	2.51E-03
CA2	2.80		2.96E-07	9.36E-05
CAMK2N1	-1.58	0.13	7.78E-06	6.96E-04
GALNT7	-0.74		2.11E-03	3.84E-02
GPC1	-0.97		5.00E-05	2.80E-03
MPDZ	1.39	0.57	1.55E-05	1.17E-03
NTN4	-2.03		3.75E-08	2.33E-05
SPARC	3.70		1.17E-10	4.87E-07
TFPI	2.55	1.55	3.56E-04	8.60E-03
TMEM98	-1.14		3.92E-05	2.50E-03

B

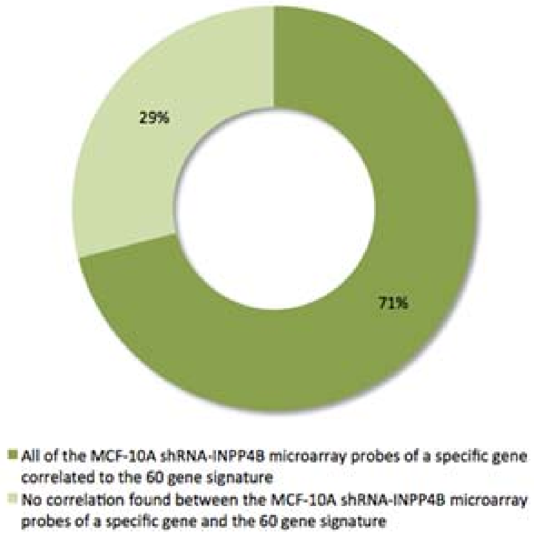

Supplemental Figure S3: Validation of 'BRCA1-negative signature' in INPP4B-knockdown cells. A. Quantitative RT-PCR of over- and under-expressed genes was conducted using RNA extracted from MCF-10A INPP4B and Renilla luciferase knockdown cell pools. Values are displayed in a bar graph as log(2)-fold change over shRNA-Renilla luciferase control and summarized in a table. RT-PCR of INPP4B knockdown cell pools displayed similar log(2) fold changes compared to corresponding microarray probes, with gene expression changes pointing in the same direction. Dark grey bars represent microarray log(2) fold changes and light grey bars represent RT-PCR log(2) fold changes in INPP4B knockdown cells. Error bars represent S.D. ($n = 3$) **B.** Bioinformatic analysis of MCF-10A shRNA-INPP4B microarray data by comparison with the 60-gene signature described in Konstantinopolous et al. (19) (see also Supplemental Table 1).

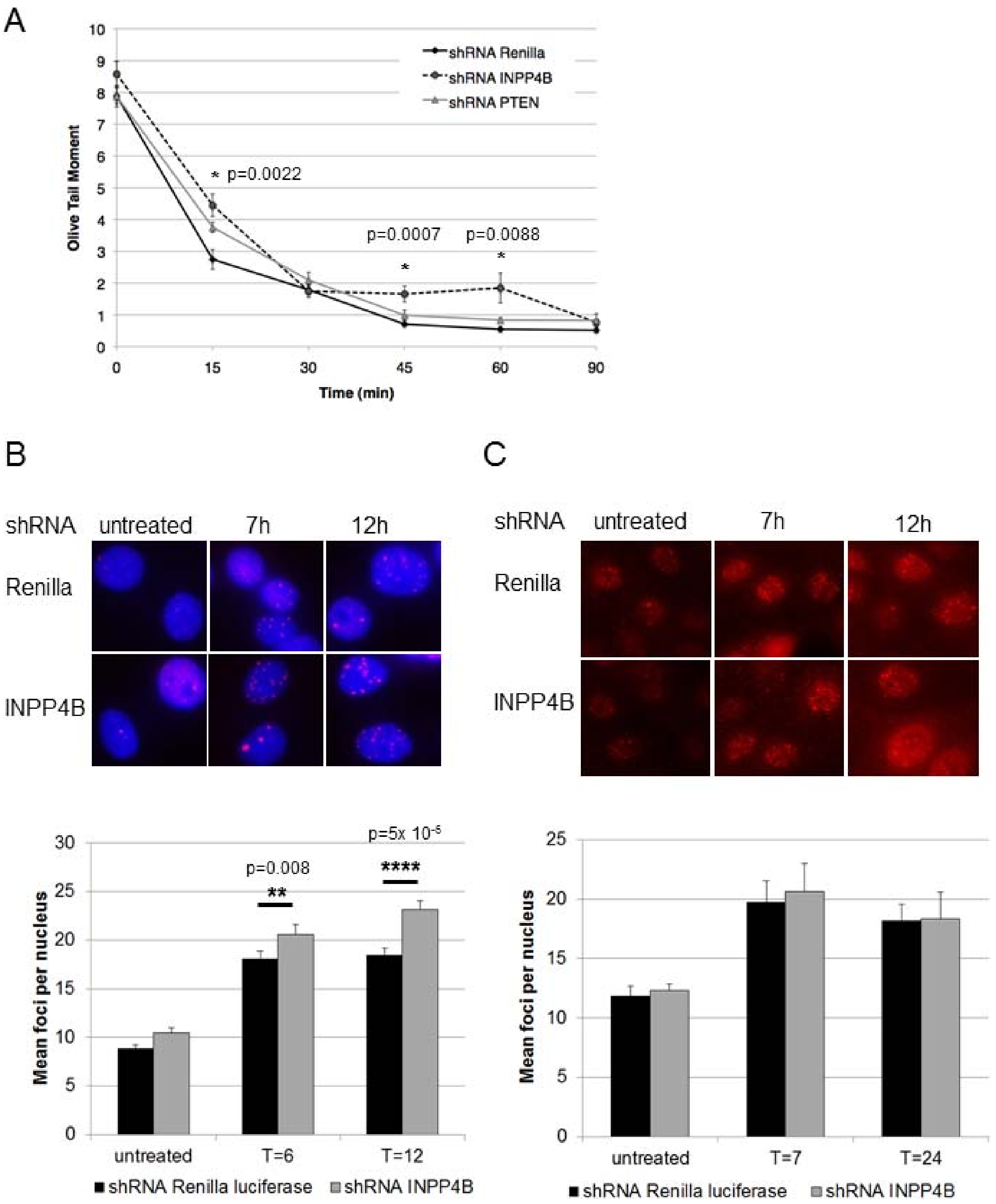

Supplemental Figure S4: DNA repair defect establishment: PTEN loss has no effect on DNA repair by comet assay, 53BP1 foci retention found in shRNA INPP4B cells and cytoplasmic-nuclear shuttling of BRCA1 is unchanged in INPP4B knockdown cell pools. A. Stable Ovca429 Renilla luciferase, INPP4B and PTEN knockdown cell pools were irradiated with 30 Gy and comet assays conducted. Tail moments were measured 0 min, 15 min, 30 min, 45 min, 60 min and 90 min post-treatment. **B.** INPP4B knockdown revealed an increase 53BP1 recruitment after etoposide treatment compared to control knockdown cell pools. Stable Ovca429 knockdown cell pools were fixed and stained for 53BP1 foci formation 6 h and 12 h after 10 µM etoposide treatment. A minimum of 100 cells per condition was quantified manually. Representative pictures of foci are displayed. **C.** Nuclear BRCA1 foci formation upon X-ray irradiation of Ovca429 knockdown cell pools was unchanged in INPP4B knockdown cell pools compared to knockdown controls after irradiation. Stable Ovca429 knockdown cell pools were x-ray irradiated (2 Gy) and cells fixed and stained for BRCA1 at time points $t = 0$ h, 7 h and 24 h. Nuclear recruitment of cytoplasmic BRCA1 was analyzed. Error bars represent S.D. ($n = 3$).

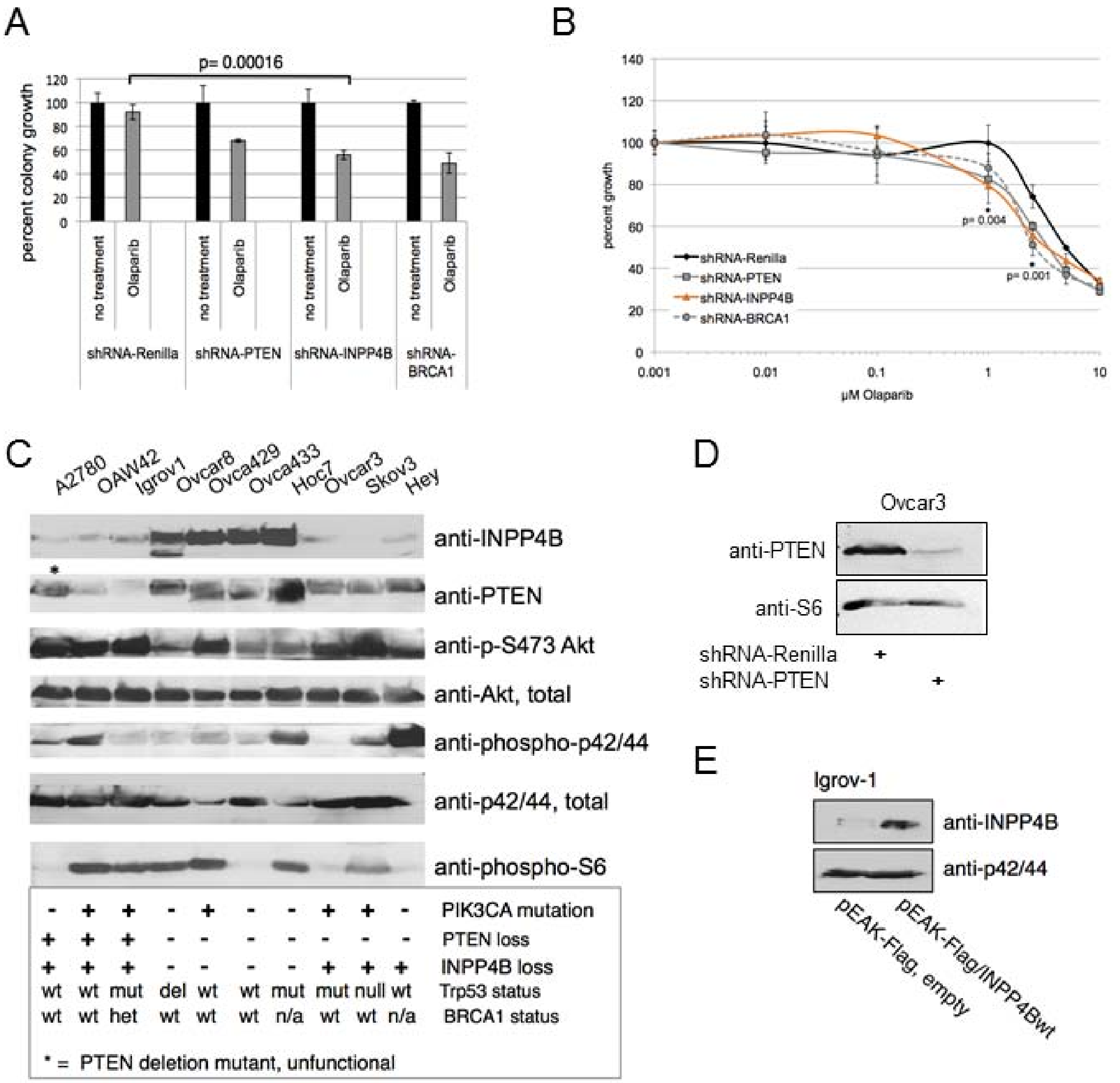

Supplemental Figure S5: PARP sensitivity and INPP4B loss *in vitro*. A. INPP4B knockdown resulted in significantly decreased colony formation ability upon PARP inhibitor treatment. The human ovarian cancer cell line Ovca429 was stably infected with hairpins directed against Renilla luciferase (control), PTEN, INPP4B and BRCA1. Cells were treated continuously with 1 µM olaparib 24 h after seeding for 5 days and then grown colonies counted. Percentage clonogenic growth of a representative experiment is displayed (error bars represent S.D., $n = 3$). **B.** INPP4B knockdown resulted in significantly reduced cell proliferation upon PARP inhibitor treatment. Ovca433 cells stably expressing shRNA hairpins directed against Renilla luciferase, PTEN, INPP4B and BRCA1 were seeded in triplicates and continuously treated with increasing concentrations of olaparib. Cell viability was measured with AlamarBlue on day 5. A representative experiment is shown ($n = 3$). **C.** Protein expression and signaling pathway activation status of 10 human ovarian cancer cell lines. Expression levels of INPP4B, PTEN, phospho-S473 AKT, phospho-p42/44 and total p42/44 are shown. The status for PI3K catalytic active mutation, PTEN and INPP4B loss as well as trp53 and BRCA1 status are displayed in the table below. **D.** Western blot analysis of human ovarian cancer cell line Ovcar3 stably expressing shRNA-Renilla luciferase or shRNA-PTEN. Cell lysates from knockdown cell pools were separated by SDS-PAGE and western blot probed for PTEN or total S6 protein (loading control). **E.** Western blot analysis of human ovarian cancer cell line Igrov-1 stably expressing pEAK-Flag/INPP4B wt or pEAK, empty vector control. Cell lysates were separated by SDS-PAGE and western blot probed for INPP4B or p42/44 protein expression.

(*Continued*)

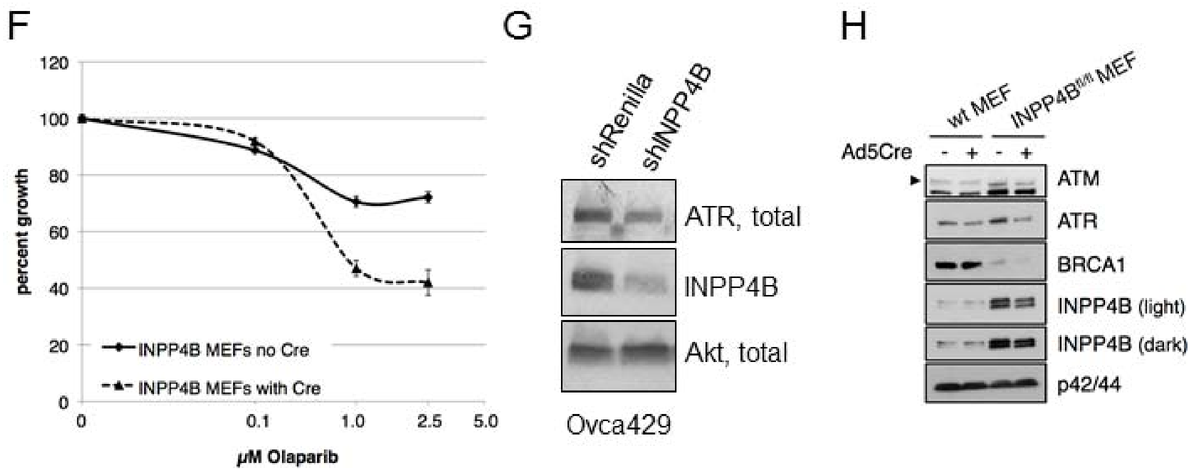

Supplemental Figure S5 (*Continued*): F. Genetic ablation of *INPP4B* in INPP4B$^{fl/fl}$ MEFs results in increased olaparib sensitivity. INPP4B$^{fl/fl}$ MEFs were infected with Adenovirus Cre recombinase (Ad5Cre) and treated with increasing concentrations of Olaparib for 5 days. Percentage cell proliferation of a representative experiment plotted (n = 3, error bars represent S.D.). **G.** Western blot analysis of human ovarian cancer cell line Ovca429 stably expressing shRNA-Renilla luciferase or shRNA-INPP4B. Cell lysates from knockdown cell pools were separated in SDS-PAGE and western blot probed for INPP4B, ATR or total Akt1/2 (loading control). **H.** Western blot analysis of AD5Cre treated wild type MEFs or INPP4B$^{fl/fl}$ MEFs. Lysates were separated in SDS-PAGE and western blot probed for INPP4B, BRCA1, ATM, ATR and p42/44 (loading control).

Supplemental Table S1. Analysis of gene expression status of INPP4B knockdown cell pools according to 60-gene signature
